# Application of machine learning methodology for pet-based definition of lung cancer

**DOI:** 10.3747/co.v17i1.394

**Published:** 2010-02

**Authors:** A. Kerhet, C. Small, H. Quon, T. Riauka, L. Schrader, R. Greiner, D. Yee, A. McEwan, W. Roa

**Affiliations:** * Department of Oncology, University of Alberta, Edmonton, AB; † Department of Radiation Oncology, Cross Cancer Institute, Edmonton, AB; ‡ Department of Medical Physics, Cross Cancer Institute, Edmonton, AB; § Department of Oncologic Imaging, Cross Cancer Institute, Edmonton, AB; || Department of Computing Science, University of Alberta, and Alberta Ingenuity Centre for Machine Learning, Edmonton, AB

**Keywords:** Positron-emission tomography, pet, radiation treatment, lung cancer, gross tumour volume, gtv, artificial intelligence, machine learning, support vector machine, svm

## Abstract

We applied a learning methodology framework to assist in the threshold-based segmentation of non-small-cell lung cancer (nsclc) tumours in positron-emission tomography–computed tomography (pet–ct) imaging for use in radiotherapy planning. Gated and standard free-breathing studies of two patients were independently analysed (four studies in total). Each study had a pet–ct and a treatment-planning ct image. The reference gross tumour volume (gtv) was identified by two experienced radiation oncologists who also determined reference standardized uptake value (suv) thresholds that most closely approximated the gtv contour on each slice. A set of uptake distribution-related attributes was calculated for each pet slice. A machine learning algorithm was trained on a subset of the pet slices to cope with slice-to-slice variation in the optimal suv threshold: that is, to predict the most appropriate suv threshold from the calculated attributes for each slice. The algorithm’s performance was evaluated using the remainder of the pet slices. A high degree of geometric similarity was achieved between the areas outlined by the predicted and the reference suv thresholds (Jaccard index exceeding 0.82). No significant difference was found between the gated and the free-breathing results in the same patient. In this preliminary work, we demonstrated the potential applicability of a machine learning methodology as an auxiliary tool for radiation treatment planning in nsclc.

## 1. INTRODUCTION

Lung cancer represents a major public health problem. *Canadian Cancer Statistics* estimated that 14% of the approximately 166,400 new cases of cancer in 2008 would be new lung cancer cases 1. Worldwide, lung cancer continues to be the leading cause of cancer-related mortality in men and women alike 2. Several potential treatments are currently available for lung cancer, including surgery, chemotherapy, and radiotherapy, but outcomes are generally poor, with a 5-year overall survival of only approximately 15% 3,4.

Current-day radical radiotherapy treatment consists of three-dimensional (3D) conformal delineation of the tumour volume based on the 3D computed tomography (ct) image. Positron-emission tomography (pet) 5–7 is already recognized as a valuable diagnostic technique in lung cancer, with higher sensitivity and specificity than ct provides 8–10; however, the role of pet in radiation treatment planning is not as well established. A number of publications have already demonstrated that including pet imaging in the process of tumour volume definition often alters the result 8–13.

The delineation of the tumour volume in tomographic images is performed by a radiation oncologist. This process is not only time-consuming, it is also prone to inter- and intra-observer variability. The development of a computerized delineation tool that would be able to assist a radiation oncologist by providing a “second reader” opinion (and possibly substituting for a radiation oncologist in the future) is therefore greatly wanted.

Several threshold-based algorithms have been proposed for the automatic delineation of lung cancer in pet images 14–19, but none of these algorithms has proved to be robust enough for routine use 11,20. The proposed algorithms suggest that the optimal suv threshold is usually a linear function of 1–2 attributes of the pet image, such as the mean suv of background tissue and the maximum suv observed in the image (SUV_max_).

In the present work, we addressed the automated delineation of lung cancer in pet images as a more complex problem that probably cannot be appropriately reflected by a *linear* combination of 1–2 attributes. Specifically, as compared with the foregoing algorithms, we proposed to base the calculation of the optimal thresholds on *richer* information (“attributes”) extracted from pet images, and to use a more flexible machine learning methodology to generate a *non-linear* dependency between the optimal thresholds and the attributes.

## 2. PATIENTS AND METHODS

Our study was approved by the research ethics board of our institution.

### 2.1 Patients and Data

We analyzed data for two patients, where each patient had both a free-breathing and a gated study. Each study comprised three images: ^18^F-fluorodeoxyglucose (^18^fdg)–pet and ct images obtained using a Philips Gemini pet/ct scanner (Philips Medical Systems, Andover, MA, U.S.A.), and a treatment planning ct image acquired on a Philips Brilliance ct scanner (Philips Medical Systems). A single bed position was used for the gated pet images (thorax area only; resolution: 144×144 voxels; voxel size: 4×4×4 mm). The free-breathing pet images were acquired using multiple bed positions covering the whole body at the foregoing resolution and voxel size. However, only the axial slices corresponding to the thorax were used for the present work. The free-breathing pet imaging started 90 minutes after 18fdg injection and was immediately followed by the corresponding gated imaging (approximately 120 minutes post 18fdg injection).

### 2.2 Data Preparation: Attributes and Reference Thresholds

For each study, the reference gross tumour volume (gtv) was identified by two experienced radiation oncologists based on the corresponding three spatially registered images (pet–ct and treatment ct). The mean suv inside the 70% SUV_max_ 3D contour was also calculated (SUV_70_).

The pet slices containing the tumour and eight adjacent tumour-free slices were extracted. Each of these pet slices was next assigned a reference suv threshold and a set of attributes: For each tumour-containing slice, the threshold that most closely approximated the corresponding gtv contour was used as the reference suv threshold. The definition of these thresholds was performed by radiation oncologists, because they take into account not only the geometric similarity, but also other criteria (anatomic information and so on). For each tumour-free slice, the maximum suv of that slice was used as the reference suv threshold.

Several articles that compared and reviewed threshold-based tumour delineation algorithms suggested that (other things being equal) contrast-oriented algorithms should be used 10,15. The algorithm proposed in Nestle *et al.* 15 defines the optimal threshold value as 0.15×SUV_70_ over the mean background suv uptake, arguing that SUV_70_ is less subject to image noise than is SUV_max_. Our observations have shown that the contours produced by thresholds lower than 0.1×SUV_70_ normally include both the tumour and the surrounding background tissue, whereas the contours produced with thresholds higher than 0.2×SUV_70_ normally only partially cover the tumour. On the other hand, some studies suggest that the optimal threshold values can vary with target volume and cross-sectional area 18. In line with the foregoing considerations, we calculated the following 6 attributes for each pet slice:

- The area and mean suv inside the 0.1×SUV_70_ contour
- The area and mean suv inside the 0.15×SUV_70_ contour
- The area and mean suv inside the 0.2×SUV_70_ contour

**FIGURE 1:**
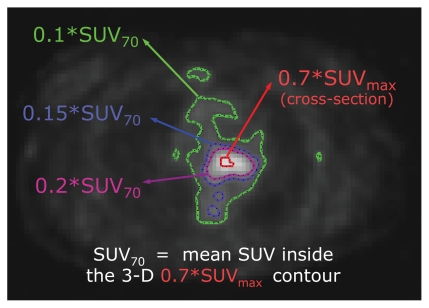
An example of the contours discussed in “2.2 Data Preparation.” Please refer to the text for more details. suv = standardized uptake value; suv_max_ = maximum suv.

Figure 1 presents an example of the foregoing contours. In other words, we propose to describe the distribution of suv in the given slice not by considering the SUV_70_ value only, but by considering the more informative interplay between the uptake and the size of the following three nested areas: the tumour and surroundings (0.1×SUV_70_ contour), approximately the tumour (0.15×SUV_70_ contour), and the hottest part of the tumour (0.2×SUV_70_ contour). Our experiments have shown that using these three contours—rather than 0.15×SUV_70_ alone—leads to a 2%–4% increase in the method’s performance.

### 2.3 Algorithm Training

**FIGURE 2:**
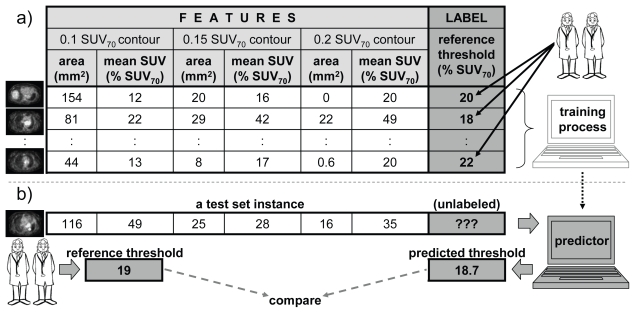
A summary of algorithm training and performance evaluation. (a) A subset of positron-emission tomography (pet) slices is used to train the algorithm. For each slice, 6 attributes (the “feature vector”) are calculated, and the reference threshold (“label”) is manually assigned by two radiation oncologists. Together, a feature vector and a label form a “labelled instance” (row in the table), and a set of such instances obtained from the selected subset of pet slices forms the “training set” (the table). During the training process, a learning algorithm uses the training set to learn how the label (the threshold) depends on the feature vector (the 6 attribute values). (b) Later, when given a new pet slice (from a “test set”), the 6 attributes are calculated and sent to the trained predictor, which returns the corresponding threshold. To evaluate the performance of the predictor, two radiation oncologists manually assign the reference threshold for this specific pet slice. Reference and predicted thresholds are then compared to evaluate the quality of the predictor. suv = standardized uptake value.

Using the machine learning terminology, our 6 attributes represent the “feature vector.” The corresponding reference suv threshold represents the dependent variable, here called the “label.” If, for some pet slice, both the feature vector and the corresponding label are known, then that features–label pair is called a “labelled instance”—that is, an instance of the relationship between the dependent variable and the features. The objective of the training process is to reflect (“learn”) this relationship from a number of labelled instances—the “training set.” Once the relationship is learned, it can be used to predict the labels for new feature vectors that are different from the ones used for training. In essence, pet slices from the training set are used to train the algorithm to predict the best threshold based on the slice attributes. Once the algorithm is trained, it can be used to predict the best threshold on new pet slices. Figure 2 summarizes this process.

The learning algorithm used for this work belongs to the family of “support vector machines” (svms) 21. These are relatively new algorithms based on the results of statistical learning theory 21, which has demonstrated excellent results in a wide range of applications. Namely, we used μ-svm for regression estimation with Gaussian kernel and with the model selection performed by a fivefold cross-validation on a logarithmic grid of hyperparameters. (Further details go beyond the scope of this journal and its audience. The interested reader is referred to Vapnik’s *The Nature of Statistical Learning Theory* 21 and Smola and Schölkopf’s “Tutorial on support vector regression” 22.) All the experiments were performed using Matlab scripts (version 7.0.1 R14: The Math-works, Natick, MA, U.S.A.) developed in house and a Matlab interface of the publicly available libsvm library (Chih-Jen Lin, National Taiwan University).

Each study of each patient was analyzed separately and independently. The pet slices were randomly divided into two groups (75% and 25% of slices). The labelled instances obtained from the first group of slices were used to form a training set, which was then used to train the algorithm. The instances obtained from the remaining 25% of slices were used to form a “test set” (hidden during the training process and preserved to evaluate the performance of the trained algorithm). This random splitting was repeated 5 times, resulting in 5 different pairs of training and test sets, each of which was used for training and subsequent evaluation of 5 different svms. The 5 evaluation results were then averaged. Table I summarizes the characteristics of the various datasets.

### 2.4 Results Evaluation

The two measures used to evaluate the results [on a test set—see Figure 2(b)] were these:

- The correlation coefficients between the reference thresholds and those predicted by the algorithm were calculated.
- The quality of the results was evaluated in terms of geometric similarity of the regions contoured with the reference thresholds, and the regions outlined by the algorithm-predicted thresholds. To this end, a Jaccard similarity coefficient was calculated:
  [1] J= |R∩A|/|R∪A|,
  where *R* and *A* stand for the regions contoured by the reference and algorithm-predicted thresholds, respectively; |*R*∩*A*| is the number of voxels that *R* and *A* have in common; and |*R*∪*A*| is the number of voxels belonging to either *R* or *A* (that is, in only *R*, or in only *A*, or in *R* and *A* together).

**FIGURE 3:**
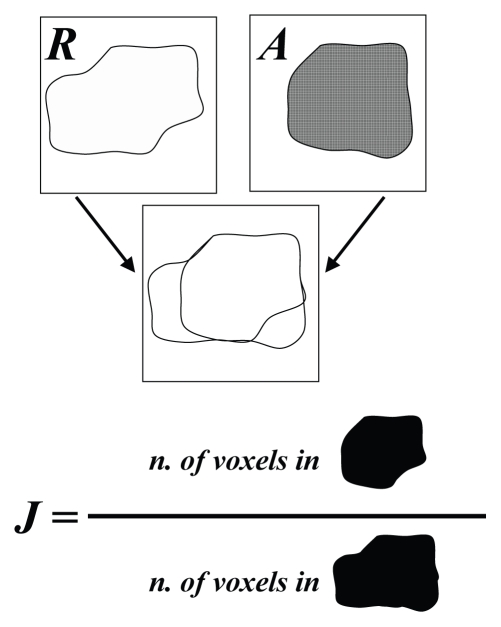
An illustration of how the Jaccard similarity coefficient (*J*) for two regions (*A* and *R*) is determined.

The Jaccard index is equal to zero when two regions have no common area and equal to unity when the regions match perfectly. Figure 3 presents an illustrative example of a Jaccard index calculation.

## 3. RESULTS

**FIGURE 4:**
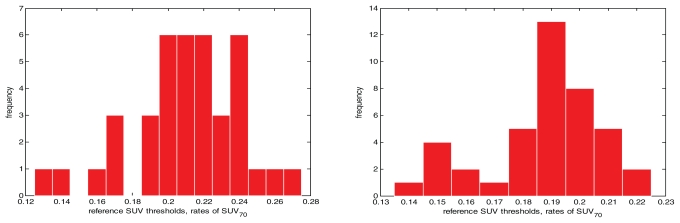
The histograms for reference standardized uptake value (suv) thresholds: patient 2, free-breathing study (left) and gated study (right).

**FIGURE 5:**
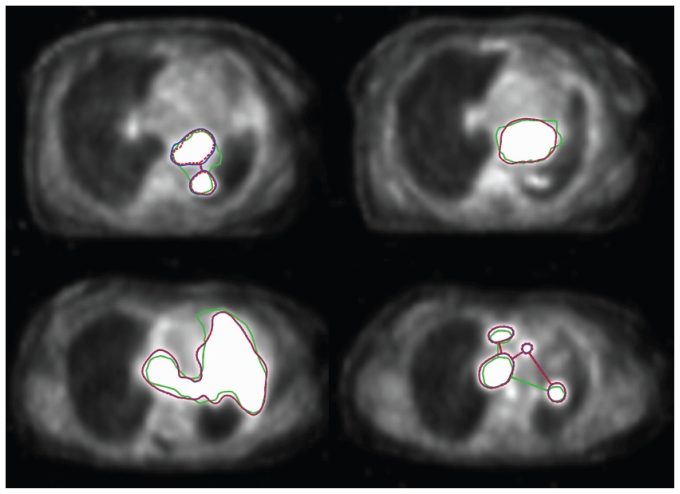
Segmentation examples in the gated pet (left: patient 1; right: patient 2). Green contour = gross tumour volume (gtv); blue contour = region contoured by the reference standardized uptake value (suv) threshold; dashed red contour = region contoured by the support vector machine–based algorithm prediction using the suv threshold.

Figure 4 shows the slice-to-slice variation of reference suv thresholds for patient 2. Table II summarizes the results obtained, and Figure 5 presents several examples of contours.

For illustrative purposes, a previously published contrast-oriented algorithm 15 was also applied to contour the tumours on the test set slices (two rightmost columns of Table II). Because of fundamental difference between that algorithm and the svm-based algorithm, these two sets of results should not be directly compared. In the present work, we applied the svm-based algorithm in an *intra-patient* fashion, with both the training set and the test set being obtained from the same pet image as described in “2. Patients and Methods.” In our approach, some knowledge about the pet image has to be provided by a radiation oncologist (in the form of the training set) to train the svm-based algorithm before the contouring proceeds. In contrast, prior knowledge of this kind is not required for the contrast-oriented algorithm.

Table II also demonstrates better results for the second patient. One of the possible explanations is that the second patient had a bigger tumour, occupying about 30% more pet slices (see Table I), resulting in a bigger training set and, hence, better training. (Learning performance typically improves with the number of instances 21.) No significant difference was found when the results of the gated and the free-breathing studies in the same patient were compared.

A single prominent peak is observable on the histogram for the gated study (Figure 4, left panel), which is not the case for the corresponding free-breathing study. This observation also holds true for the suv thresholds in patient 1. The exact mechanism of this phenomenon is unclear; it may be attributable to the presence or absence of respiratory motion or to different 18fdg post-injection times for the gated and the free-breathing studies.

## 4. DISCUSSION

The methods for pet-based gtv definition of lung cancer can be broadly divided into two groups. The first group aims to define the gtv by searching for some “inhomogeneity” throughout the pet image. Although there are some interesting examples from this group, such as gradient-based (watershed) methods 23,24 and a multimodal generalization of level set method 25, they are not as well established or as frequently cited in current reviews as are the methods from the second group. The second group aims to define the optimal suv threshold so as to delineate the gtv. These approaches include using a fixed suv (for example, 2.5) or a fixed percentage of suv_max_ (for example, 40%). Other more sophisticated contrast-oriented approaches to determining the optimal threshold include mean target suv versus mean background suv, source-to-background ratio, or the interplay of a target size and target-to-background contrast 14–19.

Our approach falls into the second group, with two important distinctions. First, the optimal suv threshold definition is based on a richer set of attributes calculated for the pet images. Secondly, we used an “adaptable” machine learning algorithm capable of approximating data in a complex nonlinear way to define the optimal suv threshold based on the established attributes.

The two threshold contours (reference and predicted) in Figure 5 look very similar; however, this similarity does not guarantee high similarity between them and the gtv. For example, both the predicted and the reference region in the upper leftmost panel are composed of two contours, whereas the corresponding gtv is a single contour, including some additional area. The explanation of this observation has two aspects: First, the radiation oncologist uses all the available material and continuously references the ct *and* the pet images during the process of gtv delineation. In contrast, a pet delineation process is based on the pet information only. Second, there is a limitation inherent in *any* approach based on suv thresholding. A radiation oncologist can assign the gtv nearly any imaginable shape, but the shape provided by any suv threshold is fixed. Therefore, choosing from a set of thresholds is equivalent to choosing from a set of fixed shapes, and sometimes (as in case of the upper leftmost panel of Figure 5), none of these fixed shapes resembles the manually drawn gtv closely enough. That is, even when performed in the best possible way, the threshold-based delineation of pet images is not necessarily sufficient, by itself, to define gtv; nonetheless, pet definition is helpful as an adjunct to target definition by the radiation oncologist.

Much effort has bseen made in this research to generate data samples of high quality, so that the results obtained could be attributed to the algorithm used rather than to some unwanted artefacts of data preparation. To this end, *three* tomographic images were thoroughly reviewed by the consensus of *two* experienced radiation oncologists for each study. This commitment to data quality (rather than quantity) and the associated time demand explain a rather moderate number of studies analyzed in the present work. We then used an *intra-patient* scenario, in which some initial input from a radiation oncologist (in the form of a training set) was required for each study to train the algorithm before it could process the remaining slices of the image. The results obtained for this intra-patient scenario encourage us to proceed further toward our ultimate goal: a standalone delineation system that will not require any initial input from a physician. This goal implies using an *inter-patient* scenario, in which an algorithm is trained on a substantial number of representative studies. As a result, the data preparation process would need to be automated. We are currently exploring these challenges and analyzing the diagnostic and radiation treatment databases available at our institution.

## Figures and Tables

**TABLE I:** Summary of data sets

Patient	Study type	*N*	*N*_+_	*N*_−_	*N*_train_	*N*_test_
1	Gated	32	24	8	24	8
	Free-breathing	27	19	8	20	7
2	Gated	41	33	8	30	11
	Free-breathing	39	31	8	29	10

*N* = total number of slices extracted for the given patient and study; *N*_+_ = number of slices containing tumour; *N*_−_ = number of tumour-free slices; *N*_train_ = number of slices used to form the training set (randomly selected from *N* slices); *N*_test_ = number of slices used to form the test set (*N* − *N*_train_ slices).

**TABLE II:** Summary of the results

Patient	Study type	Correlation (ref. vs. svm)	Jaccard (ref. vs. svm)	Jaccard (ref. vs. co)
1	Gated	0.72	0.82	0.60
	Free-breathing	0.69	0.82	0.61
2	Gated	0.77	0.96	0.73
	Free-breathing	0.86	0.96	0.81

ref. vs. svm = comparison of the reference data with the results obtained using the support vector machine–based algorithm (correlation coefficient between the threshold values, and geometric similarity coefficient between the delineated regions); ref. vs. co = comparison of the reference data with the results obtained using the contrast-oriented algorithm 15 (geometric similarity coefficient between the delineated regions).
